# Comparative Analysis of Banana Lectins rBanLec-Like and H84T-BanLec: An In Silico and In Vitro Approach

**DOI:** 10.1007/s10930-026-10326-8

**Published:** 2026-03-19

**Authors:** Guilherme Feijó de Sousa, Chrystian Nunes Gonçalves, Danillo de Oliveira Della Senta, Camila Garcia de Souza, Alice Calderipe de Lima, João Carlos Rodrigues, Maureen Legendre, David M. Markovitz, Luciano da Silva Pinto

**Affiliations:** 1 Graduate Program in Biotechnology (PPGB), Bioinformatics and Proteomics Laboratory (BioPro Lab), Technological Development Center, Federal University of Pelotas, Pelotas, Brazil; 2 Department of Internal Medicine, Division of Infectious Diseases, and Programs in Immunology, Cancer Biology, and Cellular and Molecular Biology, University of Michigan, Ann Arbor, MI 48109 USA

**Keywords:** Lectin-carbohydrate interactions, Antitumoral activity, Molecular docking, Molecular dynamics, *E. coli* expression

## Abstract

In this study, we conducted structural and comparative analyses of two recombinant banana lectins, rBanLec-like and H84T-BanLec, using both in silico and in vitro approaches. Sequence alignment showed 91.5% identity between the lectins, with differences in 12 amino acids. Structural modeling and secondary structure analysis indicated a high degree of structural similarity, with a root mean square deviation (RMSD) of 0.382 Å, suggesting that the sequence modifications did not lead to significant structural changes. Molecular docking experiments demonstrated a strong affinity of both lectins for mannose, with rBanLec-like exhibiting a lower binding free energy (− 6.6 kcal/mol) compared to H84T-BanLec (− 4.5 kcal/mol). Molecular dynamics simulations confirmed the stability of both proteins, although rBanLec-like displayed greater conformational flexibility. Expression in *Escherichia coli* showed successful production of both lectins, but rBanLec-like was detected in both soluble and insoluble fractions, whereas H84T-BanLec was exclusively obtained in the soluble fraction. Both lectins significantly inhibited the growth of colorectal adenocarcinoma cells by 62.6% and 76%, respectively. These findings contribute to the structural and functional understanding of banana lectins, highlighting their potential for biotechnological and therapeutic applications.

**Supplementary Information:**

The online version contains supplementary material available at 10.1007/s10930-026-10326-8.

## Introduction

Lectins are proteins widely distributed in nature, found in all eukaryotic organisms as well as in various species of bacteria and viruses. Recognized for their multiple biological activities, these proteins exhibit antiviral, antifungal, mitogenic, wound-healing, antitumor, and adjuvant functions [1–3]. Lectins bind to mono-, di-, and oligosaccharides, as well as glycan structures, high mannose, galactose, chitin, sialic acid, and fucose, among others [4].

Based on specific recognition capability, lectins have shown promising results in experimental approaches for the treatment of several diseases, including different types of cancer [1, 5, 6]. Abnormal glycosylation has been identified as a mediator of a wide range of processes related to tumor development, ranging from cell adhesion to invasion, metastasis, immune response, and cancer metabolism [7, 8].

A family of lectins extensively studied in the literature, with considerable importance for medical research, is the "Jacalin-Related Lectins" (JRLs). Among them, the lectin extracted from bananas (*Musa acuminata*) stands out due to its specificity for high mannose and glucose. With an approximate molecular weight of 15 kDa, this lectin can be found in both dimeric and tetrameric forms [9, 10]. Previously, our research group developed a new banana lectin called rBanLec-like, based on the BanLec sequence. This sequence was aligned with those of nine other JRL lectins, including the *Heltuba* lectin. Based on this multiple sequence alignment, the BanLec sequence was modified by altering 11 conserved amino acids found in other JRL lectins but differing from native BanLec [11]. The amino acid substitutions aimed to create a new sequence distinct from BanLec while preserving its structure and biological activity, with the potential for increased functionality. In parallel, a research group from the University of Michigan also developed a variant of the banana lectin called H84T-BanLec. In this version, a mutation was introduced in the sugar-binding site of BanLec, reducing the protein's mitogenicity without compromising its activity or mannose-binding [12]. This modification was achieved by substituting a single amino acid in the BanLec sequence, replacing histidine with threonine at position 84, leading to the designation H84T-BanLec [12, 13]. H84T-BanLec has shown promising activity against all influenza and corona viruses tested [14, 15]. H84T-BanLec has also been used in place of an antibody derivative to construct a chimeric antigen receptor (H84T-CART -cells) that targets and kills pancreatic tumor cells and disrupts tumor-associated stroma [6].

In order to understand how these new BanLec-derived lectins (rBanLec-like and H84T-BanLec) relate to each other in terms of their specific activity against a tumor cell line (HT-29), we performed growth inhibition assays on these cells. Furthermore, using bioinformatics tools, we explored the effect of mutations on the maintenance of the structures of each lectin and correlated them with the effects observed in the target cell line. Our findings speak to the applicability of molecular engineering of lectins for therapeutic purposes.

## Materials and Methods

### Expression of rBanLec-Like and H84T-BanLec Proteins

To produce recombinant proteins, the plasmids pET-28a:H84T-BanLec and pAE: rBanLec-ike were used. The plasmids were obtained previously [11, 12] and kindly provided by the authors. The vectors were transformed into *Escherichia coli* BL21(23)—Codon-Plus RIL® and the production of the recombinant protein followed the methodology previously described by these authors.

For protein extraction, the cells were solubilized in lysis buffer (NaH₂PO₄ 20 mM, NaCl 200 mM, Imidazole 40 mM, β-mercaptoethanol 1 mM, pH 7.4) and sonicated. Centrifugation of the lysate (12,000 × g, 40 min, 4 °C) separated soluble and insoluble fractions, with the insoluble fraction treated with the same buffer containing 8 M urea. Both fractions were analyzed by SDS-PAGE to assess the solubility of the recombinant proteins. Purification was performed by metal ion affinity chromatography (IMAC/Ni^2^⁺) on a His-Trap FF column using the AKTA-purifier system. The proteins were eluted with an imidazole gradient (500 mM). The purified proteins were dialyzed against water and lyophilized for storage. The proteins were confirmed by western blot using the anti-histidine tag peroxidase-conjugated antibody (Sigma-Aldrich).

### Culture Cell and Cytotoxicity Assay

For cytotoxicity assays, HT-29 cell line (human colorectal adenocarcinoma) cells were obtained from the Rio de Janeiro Cell Bank (BCRJ Code: 0111) and cultured in Dulbecco's medium (D-MEM) supplemented with 10% FCS. The cells were maintained at 37 °C in a humidified atmosphere with 5% CO_2_ and 95% air. For cytotoxicity assays, the cells were removed from the cell culture flask by the trypsinization process (detachment and individualization of cells by enzymatic process using trypsin) and cultured in 96-well plates in a humid incubator at 37 °C with 5% CO_2_ until the establishment of a cell monolayer (approximately 3 × 10^4^ cells/mL) per well. After 24 h, cells were exposed to different concentrations of rBanLec-like, H84T-BanLec, and D133G-BanLec (negative control) [12] at different exposure times (2 h, 72 h, and 96 h). After treatment, the MTT assay was conducted according to [16]. The results represent the mean ± standard error of the mean (SEM) of three independent experiments.

### Statistical Analysis of In Vitro Tests

The cell viability values obtained in the cytotoxicity tests using the MTT assay were expressed as mean ± standard deviation (SD). Analysis of variance (ANOVA) was applied, followed by Tukey's post hoc test for multiple comparisons, using GraphPad Prism software version 7.0®. Three factors were considered: the difference in activity between the lectins, the concentration, and the time required for their action.

### In Silico Structural Analysis of Lectins

#### Comparison Between Sequences

To analyze the similarity between the sequences of the rBanLec-like and H84T-BanLec lectins, an alignment was performed using the EMBOSS Needle tool—Pairwise Sequence Alignment (PSA) (https://www.ebi.ac.uk/jdispatcher/psa/emboss_needle).

#### Comparison of the Three-Dimensional Model and Secondary Structure

The three-dimensional structure of the rBanLec-like lectin was generated through structural modeling using AlphaFold2 software, accessible via ColabFold [17]. The structure of the H84T-BanLec lectin was obtained from the Protein Data Bank (PDB), under the PDB ID: 4PIU. To compare the structures, both lectins were visually analyzed and superimposed using PyMol software (PYMOL MOLECULAR GRAPHICS SYSTEM, Schrödinger, version 2.5.4). The quality of the models was assessed with ModFold9 software [18] and MolProbity [19]. The secondary structure of the proteins was predicted with SOPMA (https://npsa-prabi.ibcp.fr/cgi-bin/npsa_automat.pl?page=/NPSA/npsa_sopma.html).

#### Molecular Docking

To analyze the interaction profile with mannose and high mannose in both proteins, molecular docking was performed. The models of proteins were prepared using the MGLTools software to remove water molecules and adjust metal ions and cofactors. The mannose and high mannose ligands were obtained from the GlyConnect database (v1.2.0, Swiss Institute of Bioinformatics—ExPASy) and minimized 3D structures were obtained using the GLYCAM-Web software. To identify the ligand binding sites in the protein structures, the FTSite web server (https://ftsite.bu.edu/) and information available in the literature were used. For molecular docking, the AutoDock Vina (v. 1.2.0) was used. A grid box was prepared in the Autodock tools program (part of the MGLtools package (v. 1.5.7), Molecular Graphics Lab) in dimensions that encompassed the carbohydrate recognition sites (Appendix 1—Supplementary Material). The interaction between the lectins and the ligand was visualized with the Pymol.

#### Molecular Dynamics

Molecular dynamics allows for the high-precision simulation of atomic movement in structures. In this study, we performed simulations of the three-dimensional structure of the two lectins in the absence of ligands, using the GROMACS tool [20]. Initially, the structures were prepared by generating a topology based on well-established and reproducible simulation protocols in CHARMM-GUI (https://www.charmm-gui.org/). A rectangular box (distance from ends = 10) was constructed with a TIP3P water model. The molecule was solvated, with 33 K + ions and 35 Cl- ions added (pH 7, 37 °C). For the dynamics execution, the CHARMM36e force field was used, and simulations were conducted with a time step of 2 fs, followed by a 6 ns equilibration process. The production phase of the molecular dynamics simulation was carried out over 100 ns.

## Results

### Production in *Escherichia coli*

**Fig. 1:**
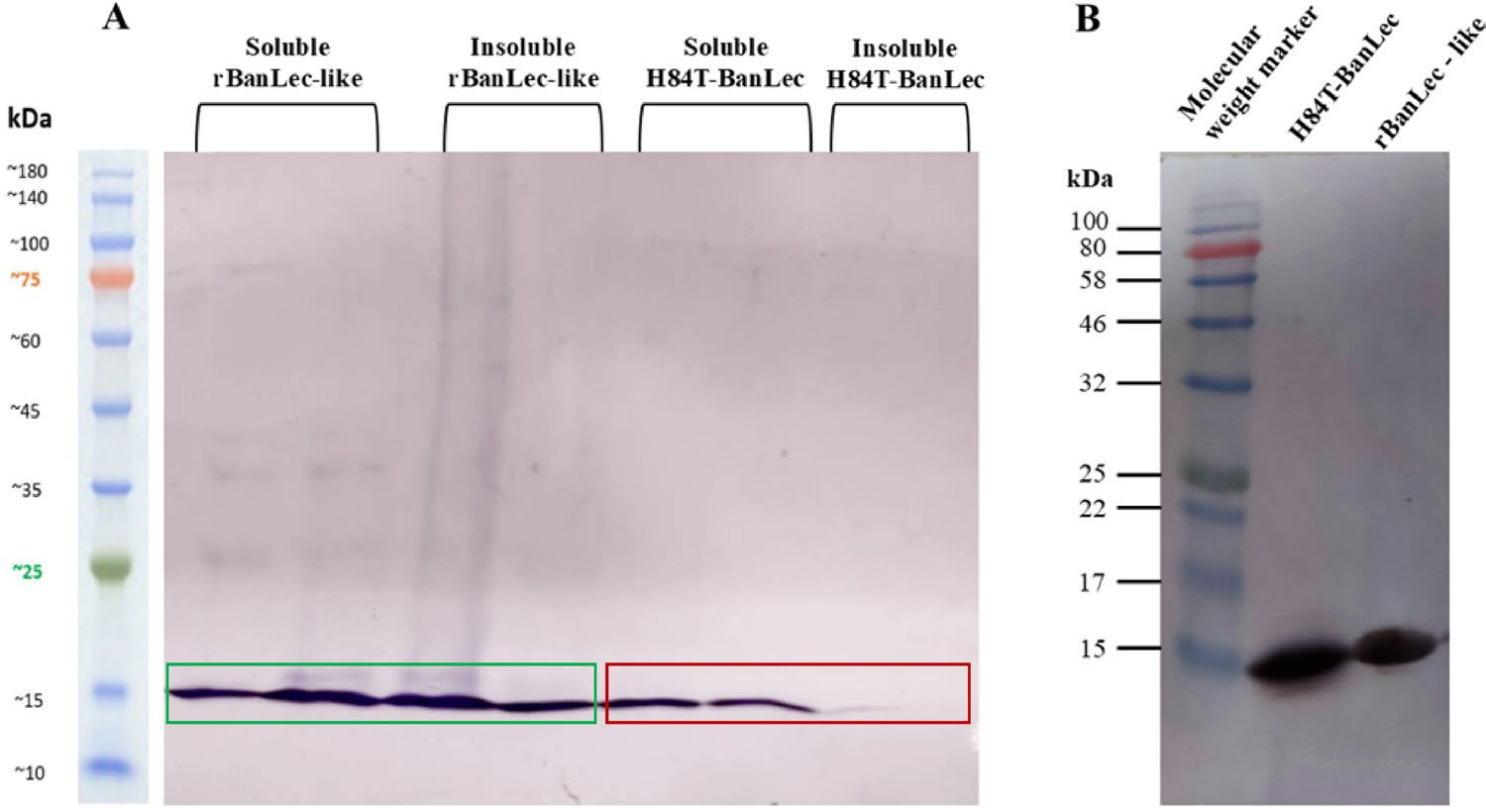
SDS-PAGE (15%) stained with Coomassie Blue (**A**) and Western blot analysis (**B**) using an anti-histidine antibody conjugated with peroxidase, illustrating the solubility profile and expression of recombinant lectins in Escherichia coli BL21 (DE3)-CodonPlus RIL®. In panel A, purified protein fractions obtained from soluble and insoluble extracts are shown. The bands highlighted in green correspond to rBanLec-like, which is detected in both soluble and insoluble fractions, whereas the bands highlighted in red correspond to H84T-BanLec, which is detected exclusively in the soluble fraction. No band is observed in the insoluble fraction of H84T-BanLec, indicating its improved solubility upon expression in *E. coli*. The absence of additional *E. coli* protein bands reflects the efficiency of the purification procedure. Panel B shows the Western blot confirmation of purified H84T-BanLec and rBanLec-like proteins. Molecular weight markers (kDa) are indicated

To compare the two lectins, they were first expressed in *E. coli.* Solubility assays for both lectins were evaluated using SDS-PAGE (Fig. 1A) and the purified lectins by western blot (Fig. 1B). The lectins rBanLec-like and H84T-BanLec were efficiently expressed, exhibiting the expected molecular mass of approximately 15 kDa, in agreement with the literature [11, 12]. Notably, rBanLec-like was found in both soluble and insoluble fractions, while H84T-BanLec was obtained exclusively in the soluble fraction. Finally, the proteins were quantified using the colorimetric method Pierce™ BCA Protein Assay Kits (Thermo Scientific™—Catalog number 23227) with a final concentration of approximately 630 μg/mL for rBanLec-like and 710 μg/mL for H84T-BanLec.

### rBanLec-Like and H84T-BanLec Promote Selective Cytotoxicity Effects on the Human Colorectal Adenocarcinoma Tumor Cell Line (HT-29)

**Fig. 2:**
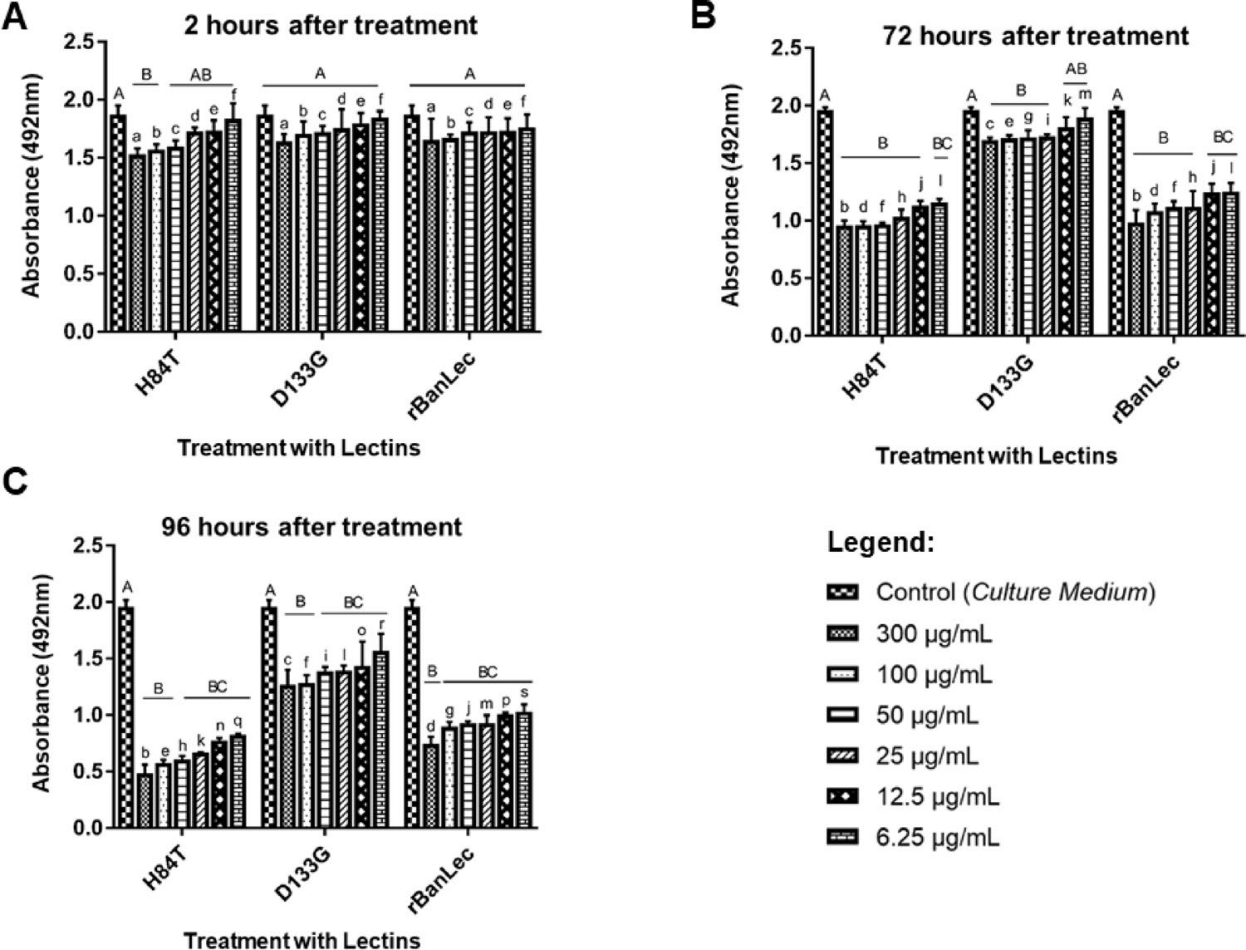
Evaluation of cell death by the MTT assay of the HT-29 cell line in relation to the banana lectins H84T-BanLec, D133G-BanLec and rBanLec-like in periods of 2 h (**A**), 72 h (**B**) and 96 h (**C**), at concentrations of 6.25 μg/mL, 12.5 μg/mL, 25 μg/mL, 50 μg/mL, 100 μg/mL and 300 μg/mL, with D-MEM as control. ANOVA followed by Tukey's post hoc was used for statistical analysis. For all p < 0.05. Different letters demonstrate significant difference. Uppercase letters represent differences within each group. Lowercase letters represent concentration differences between groups

Figure 2 shows the results of the cytotoxicity assay in the HT-29 tumor cell line after treatment with rBanLec-like, H84T-BanLec, and D133G-BanLec lectins. The D133G-BanLec protein was used as an additional control group, since it has limited biological activity [12]. A statistically significant difference was observed 2 h after treatment in the group treated with H84T-BanLec compared to the untreated control (p < 0.05) (Fig. 2A). Even with a shorter incubation time, the concentrations of 50, 100, and 300 µg/mL already showed potential to inhibit cell growth in the group treated with H84T-BanLec (14.95%, 16.40%, and 18.83% inhibition, respectively). In contrast, D133G-BanLec and rBanLec-like did not differ significantly from the control group during this same period.

After 72 and 96 h of incubation (Fig. 2B and C), all experimental groups showed significant differences compared to the untreated control (cell + D-MEM) (p < 0.05). At 72 h, D133G-BanLec exhibited lower cytotoxicity than H84T-BanLec and rBanLec-like at equivalent concentrations (indicated by different uppercase letters). Within each lectin treatment, the highest concentrations (100 and 300 µg/mL) differed significantly from the lower ones (6.25 to 50 µg/mL), as indicated by different lowercase letters. At 96 h, all concentrations differed significantly among the three lectins. As expected, H84T-BanLec strongly inhibited HT-29 cell proliferation and induced cell death by approximately 76% (Supplementary Tables S3–S5) at 300 µg/mL, while rBanLec-like inhibited growth by 54.75% at 100 µg/mL and 62.6% at 300 µg/mL (Supplementary Tables S3–S5). The negative control D133G-BanLec induced only minimal effects on cell death, showing lower inhibitory activity compared to H84T-BanLec and rBanLec-like. These statistical groupings, represented by uppercase and lowercase letters, demonstrate both the differences among lectins and the dose-dependent response observed within each treatment.

**Fig. 3:**
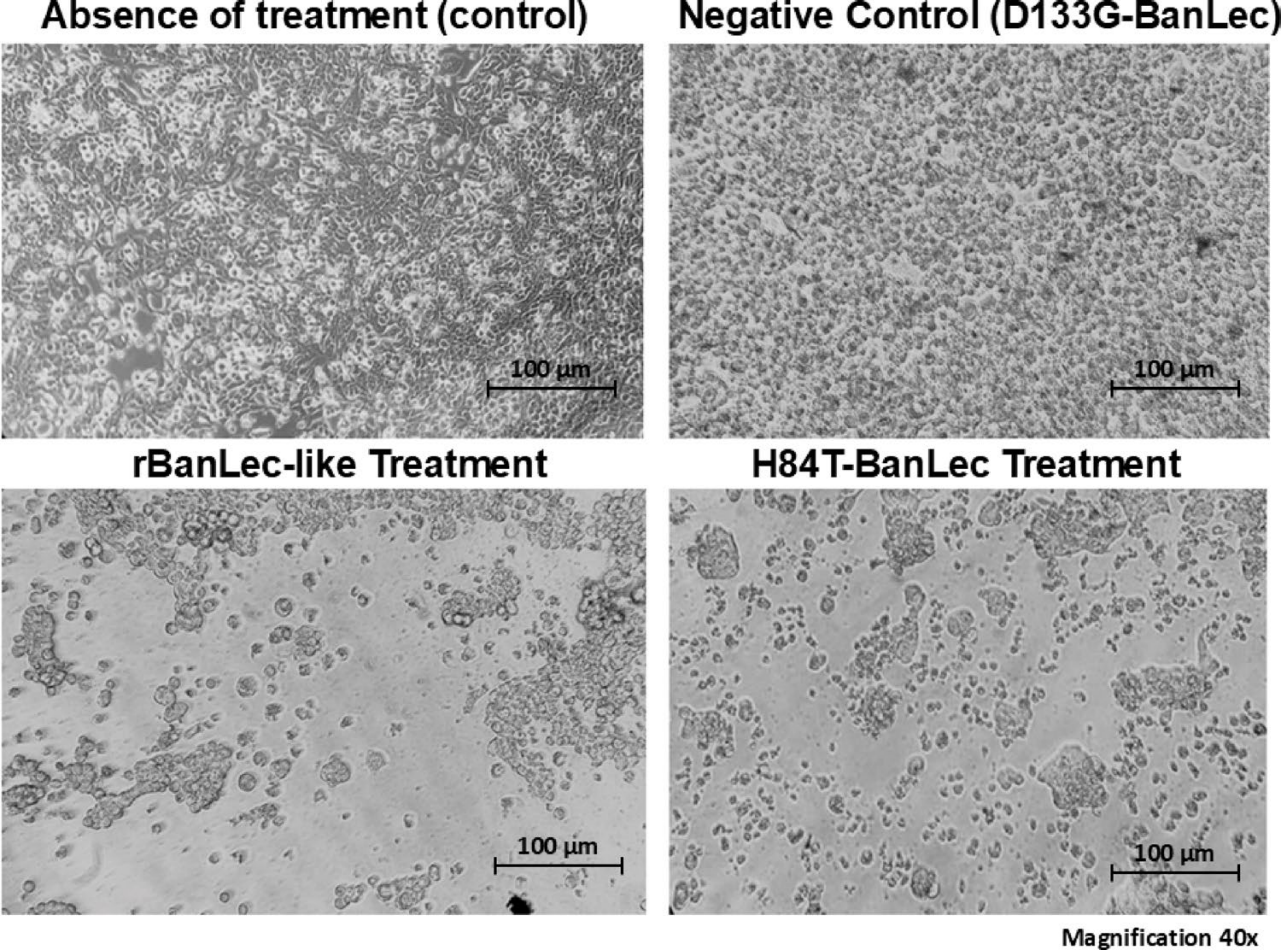
Morphological analysis of HT-29 cells after lectin treatment. Representative microscopy images of HT-29 cells under the following conditions: untreated control (no treatment), negative control (D133G-BanLec), rBanLec-like treatment, and H84T-BanLec treatment. Cells were incubated for 96 h with lectins at a concentration of 300 µg/mL. Images were acquired with a 40 × objective. Scale bars correspond to 100 µm in all panels

Analysis of the morphology of HT-29 cell lines revealed that, after 96 h of incubation with rBanLec-like and H84T-BanLec lectins at a concentration of 300 µg/mL, a change in cell shape occurred, characterized by rounding. This observation may indicate modifications in the cytoskeleton and the formation of openings in the cell monolayer, as illustrated in Fig. 3. These are typical signs of the cell death process. In contrast, the cells in the untreated and D133G-BanLec control groups maintained the typical morphology of the cell line, presenting an elongated shape and several projections in the cytoplasmic membrane, which reflect cell interaction through adhesion points (Fig. 3).

### Alignment of Sequences and Comparison of the Three-Dimensional Model and Secondary Structure of the rBanLec-Like and H84T-BanLec Lectins

**Fig. 4:**
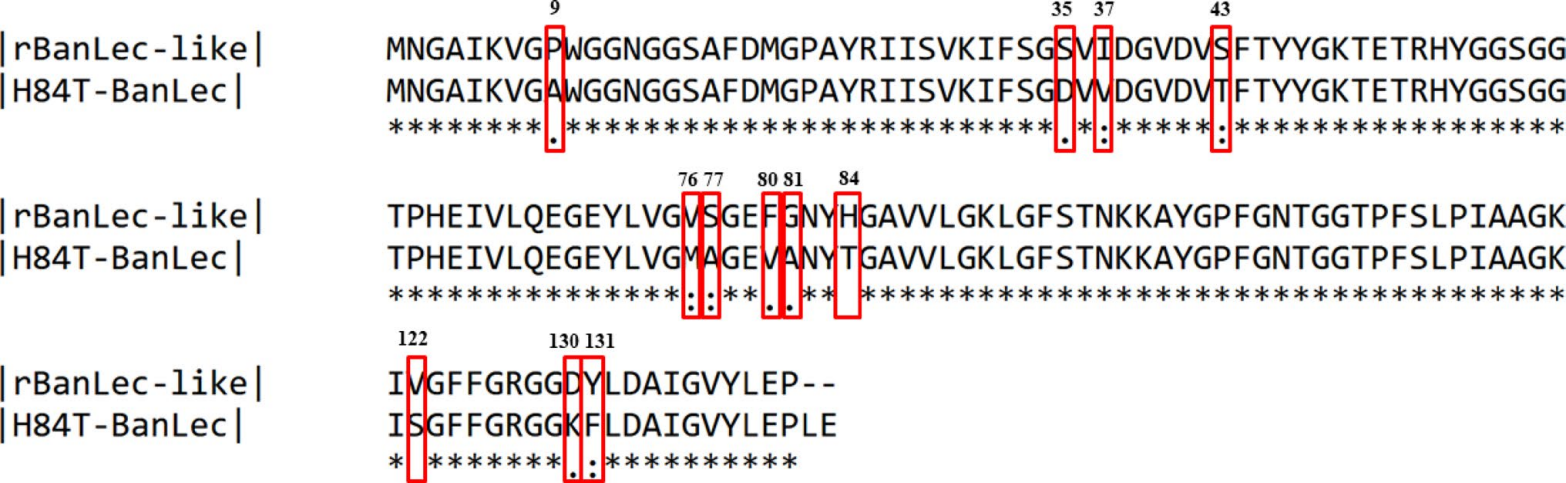
Alignment of the rBanLec-like and H84T-BanLec lectins using the Pairwise Sequence Alignment (PSA) method. The amino acid differences are highlighted in red. The numbers above the sequences indicate the position of the residues where amino acid differences occur

The amino acid sequences of the rBanLec-like and H84T-BanLec lectins were compared allowing for the identification of differences in amino acid composition between them. Figure 4 shows the sequence alignment, highlighting the residues that were modified in each protein.

Twelve differences were identified between rBanLec-like and H84T-BanLec, resulting in 91.5% identity and 93.7% similarity. The variations in residues occurred at positions 9, 35, 37, 43, 76, 77, 80, 81, 84, 122, 130, and 131, with the following substitutions observed: Pro → Ala, Ser → Asp, Ile → Val, Ser → Thr, Val → Met, Ser → Ala, Phe → Val, Gly → Ala, His → Thr, Val → Ser, Asp → Lys, and Tyr → Phe (rBanLec-like → H84T-BanLec).

**Fig. 5:**
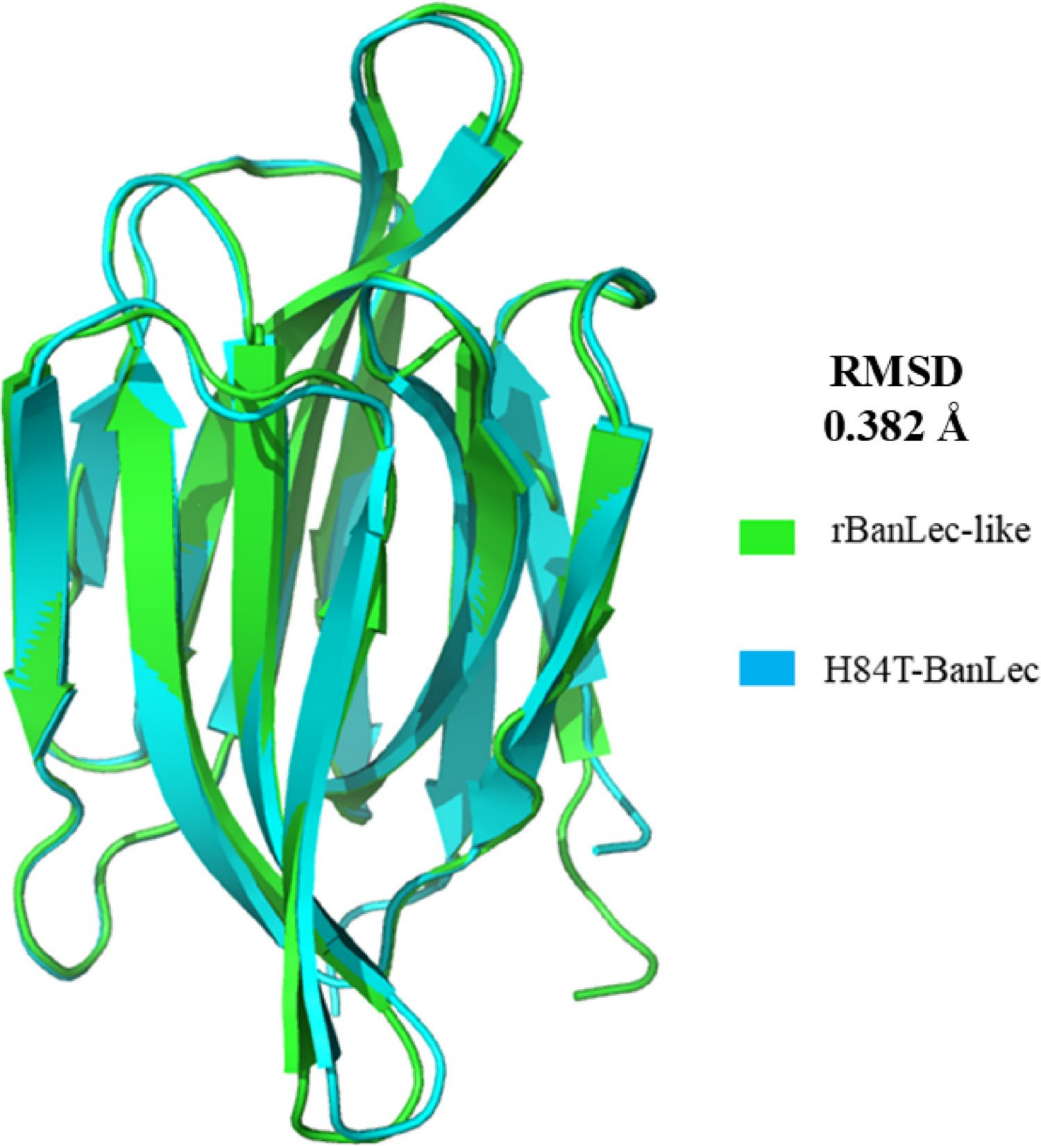
Structural superposition of the rBanLec-like lectin (green) and H84T-BanLec (blue)

To understand how changes in the sequences of the two lectins affect expression and activity outcome, the structural differences between the lectins were analyzed. The superimposition of the 3D structures (Fig. 5) of the two proteins showed an RMSD score of 0.382 Å, indicating high structural similarity. Stereochemical validation of the quality of the 3D lectin models was performed, and the rBanLec-like lectin model achieved an overall score of 0.9432, while the H84T-BanLec model presented a score of 0.9157, demonstrating high confidence in the prediction of the 3D structures of these models (Supplemenatry Material—Table S1). The p-value for rBanLec-like was 3.037E-5 and for H84T-BanLec it was 4.152E-5. Scores above 0.4 typically indicate reliable, complete models with high similarity to the native structure. An extremely low p-value confirms the statistical reliability of the model, ruling out random generation [18].

Complementary to this, the pLDDT (Predicted Local Distance Difference Test) analysis, which ranges from 0 to 100—with values above 80 indicating high confidence in structural prediction showed that most residues scored above 80 in both proteins, suggesting highly reliable models. Minor drops in confidence are likely associated with flexible regions such as loops or the N- and C-terminal (Supplementary Material—Figure S1A and S1B). Notably, the binding site residues are well-modeled, which ensures the absence of excessive flexibility that could compromise ligand interaction. This is particularly relevant, as poorly modeled regions or those with high conformational mobility may lead to inaccurate predictions of binding affinity and mode. The observed rigidity in this site suggests a higher reliability in the positioning of key residues, increasing confidence in molecular docking results and in functional analyses based on this model. The Residual Error plot (in Ångströms) showed values predominantly below 2 Å in both proteins, indicating high structural accuracy (Supplementary Material—Figure S1C and S1D) [21, 22].

**Fig. 6:**
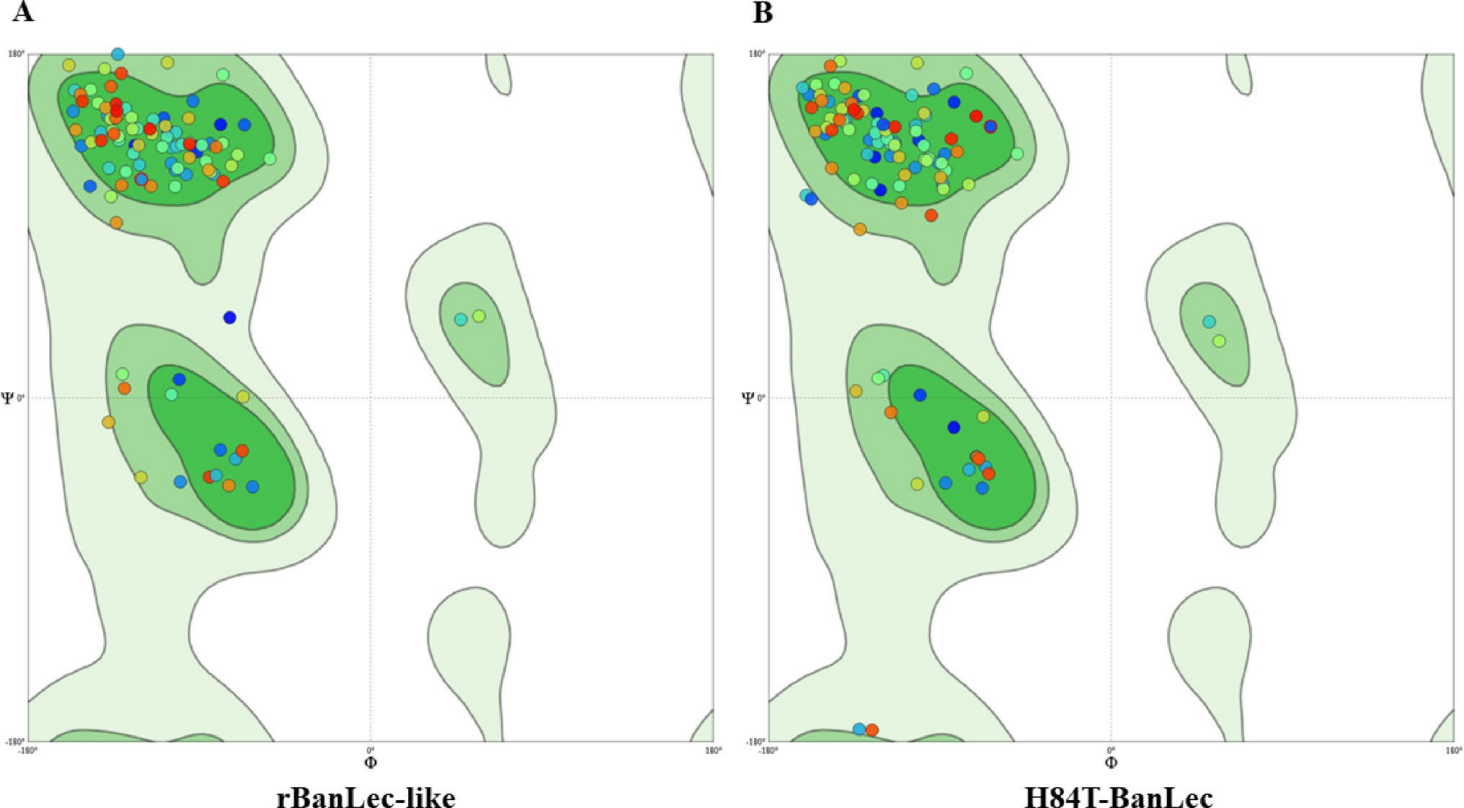
Ramachandran plots for rBanLec-like (**A**) and H84T-BanLec (**B**) lectins. The background color gradient represents the allowed conformational regions based on the torsion angles φ (phi) and ψ (psi): dark green indicates the most energetically favored regions, light green corresponds to the allowed regions, and very light green represents unfavorable or non-allowed regions. Individual amino acid residues are shown as colored dots

For a more reliable comparison between the structures, the quality of the models was also evaluated based on the generated Ramachandran diagram and by analyzing the torsions of the phi (φ) and psi (ψ) angles. For a high-quality model, at least 90% of the amino acids should be in favored regions [23, 24]. The lectins rBanLec and H84T-BanLec presented values of 96.40% and 97.12%, respectively, indicating that many residues in both lectins are in the favored regions of the graph and that the structures are well folded and energetically stable (Fig. 6).

The secondary structure analysis indicated at both lectins are predominantly composed of coil regions, followed by β-sheets and α-helices. H84T-BanLec exhibits a lower proportion of α-helices and a higher proportion of coils compared to rBanLec-like, suggesting increased structural flexibility. The β-sheets, which are similar in both proteins, indicate the conservation of the characteristic structural motif of the Jacalin family—the "β-prism" or "β-sandwich"—which forms specific binding pockets that facilitate carbohydrate recognition. (Supplementary Material—Table S2 and Figure S2).

### Molecular Docking Analysis

**Fig. 7:**
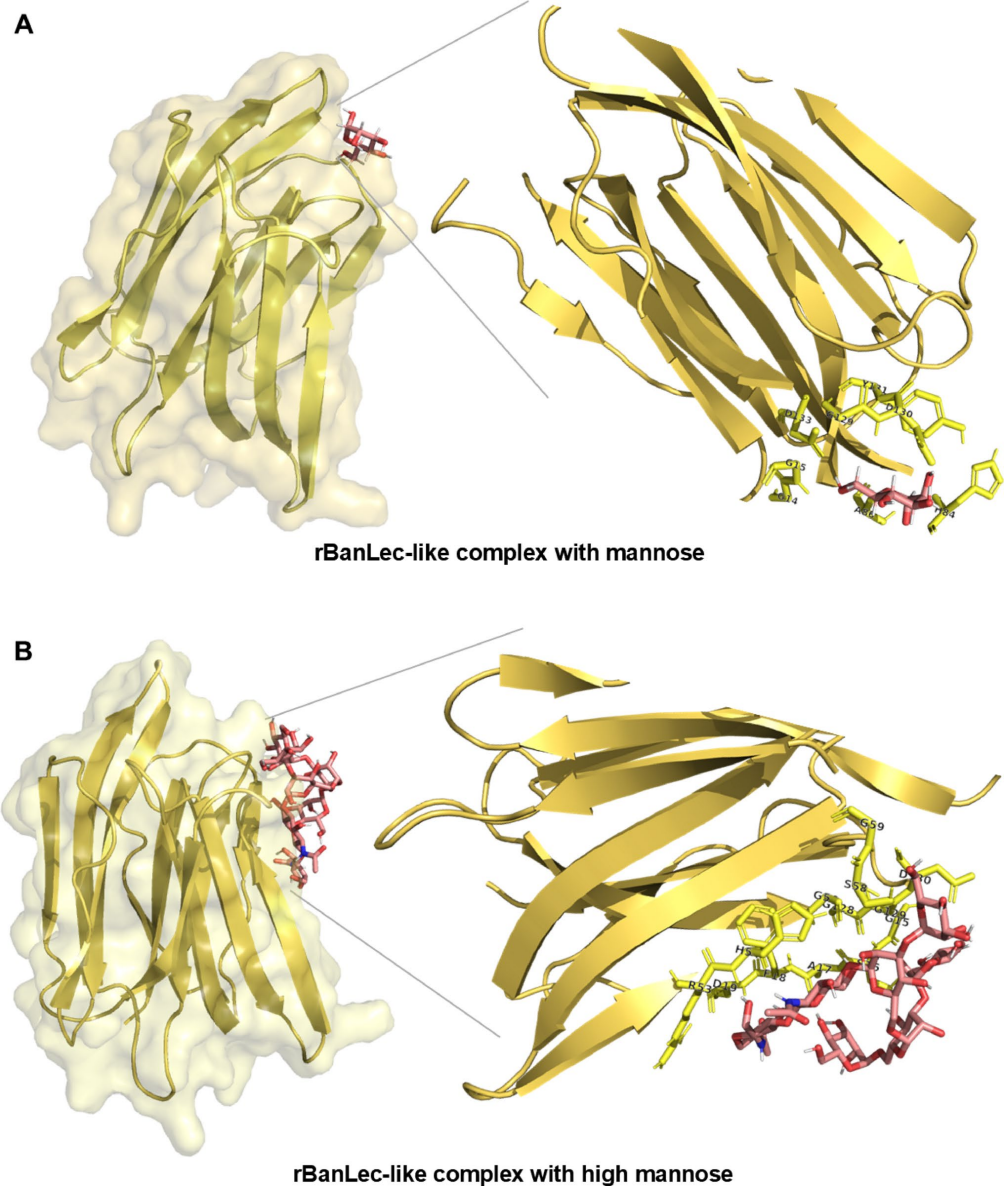

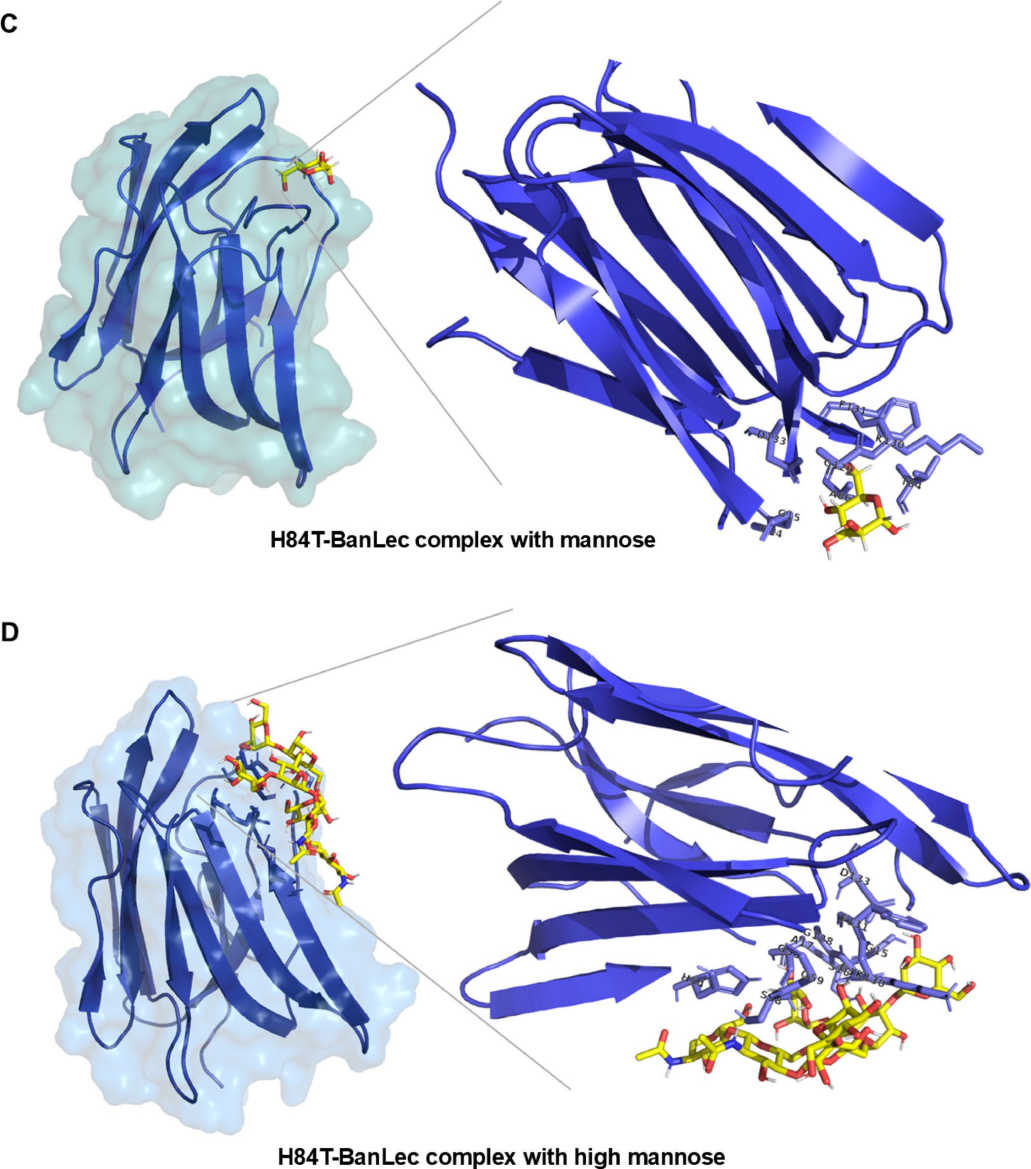
Molecular docking represents lectin interactions with mannose and high-mannose structures. In (**A**, **B**), the structure of the rBanLec-like lectin (in yellow) is shown complexed with mannose (**A**) and with a high-mannose structure (**B**). In (**C**, **D**), the structures of the H84T-BanLec lectin (in blue) are shown complexed with mannose (**C**) and with high mannose (**D**). Protein surfaces are displayed with transparency to highlight the binding site. Mannose and high-mannose ligands are shown in stick representation, illustrating the differences in site recognition and occupancy between the two lectins and the two types of ligands

After validating the structural quality and reliability of the three-dimensional lectin models, molecular docking analyses were performed to investigate their interactions with specific carbohydrate ligands. The monosaccharide D-mannose and the high-mannose structure were used as ligands. The results showed strong interactions between both lectins and carbohydrates, predominantly mediated by hydrogen bonds (Table 1). These interactions indicate a specific recognition of the ligands by the lectin binding sites, suggesting a high degree of affinity and stereochemical complementarity between the active site residues and the functional groups of the carbohydrates (Fig. 7). This molecular recognition likely contributes to the stability of the lectin-carbohydrate complexes and may be associated with their biological roles.

**Table 1:** Binding affinity values (kcal/mol) and interacting residues identified in the molecular docking analysis between D-mannose or high-mannose and the lectins rBanLec-like and H84T-BanLec

Protein	Mannose		High Mannose	
	Binding Afinnity (kcal/mol)	Residues	Binding Affinity (kcal/mol)	Residues
rBanLec-like	− 6.6	Gly14, Gly15, His84, Ala86, Val88, Gly129, Asp130, Tyr131, Asp133	− 5.4	Gly14, Gly15, Ser16, Ala17, Phe18, Asp19 Arg53, His54, Gly 56, Ser58, Gly 59, Phe104, Gly105, Asn106, Gly 128, Gly129, Asp130, Tyr 131
H84T-BanLec	− 4.5	Gly14, Gly15, Thr84, Ala86, Val88, Gly129, Lys130, Phe131, Asp133	− 6.0	Gly14, Gly15, Ser16, Ala17, Phe18, Asp38, Thr52, His54, Gly56, Ser58, Gly59, Gly128, Gly129, Lys130, Phe131, Ile132, Asp133
*wtBanLec Musa acuminata (*3MIT)*	Ser33, Gly34, Asp35, Val36, Val37, Asp38, Gly59, Gly60, Thr61, Pro62, His63, Val86 Phe104, Gly105, Asn106, Gly129, Asp130, Phe131, Ile132, Asp133			

*The binding sites of the native banana lectin from *Musa acuminata* (PDB ID: 3MIT) complexed with D-mannose were used as a reference for comparison with the molecular docking results obtained for the recombinant lectins rBanLec and H84T-BanLec

The evaluation of the mannose and high mannose docking poses with the lectin was performed using the Binding Affinity Energy (kcal/mol) as a scoring function. The poses with the best energy was selected for each docking simulation [25, 26]. Table 1 presents the results of the molecular [27] docking analysis between the lectins rBanLec-like and H84T-BanLec with the carbohydrates D-mannose and high-mannose. The binding affinity values showed that rBanLec-like exhibited a higher affinity for D-mannose (− 6.6 kcal/mol), while H84T-BanLec showed a stronger interaction with high-mannose (− 6.0 kcal/mol).

Several residues are conserved among the lectins analyzed, including Gly14, Gly15, Val88, Gly129, Asp130, and Asp133, indicating critical regions involved in carbohydrate recognition. In rBanLec-like, the presence of His84 appears to contribute to its higher affinity for D-mannose. The substitution of His84 by Thr84 in H84T-BanLec likely alters the network of hydrophobic and polar interactions within the binding site, favoring the docking and interaction of high-mannose structures. This is supported by the observation that His84 in rBanLec-like interacts directly with D-mannose. This analysis highlights how subtle changes in protein structure can modulate ligand-binding properties, which is particularly relevant for the rational design of lectins with therapeutic potential.

### Molecular Dynamics Simulation Analyses

**Fig. 8:**
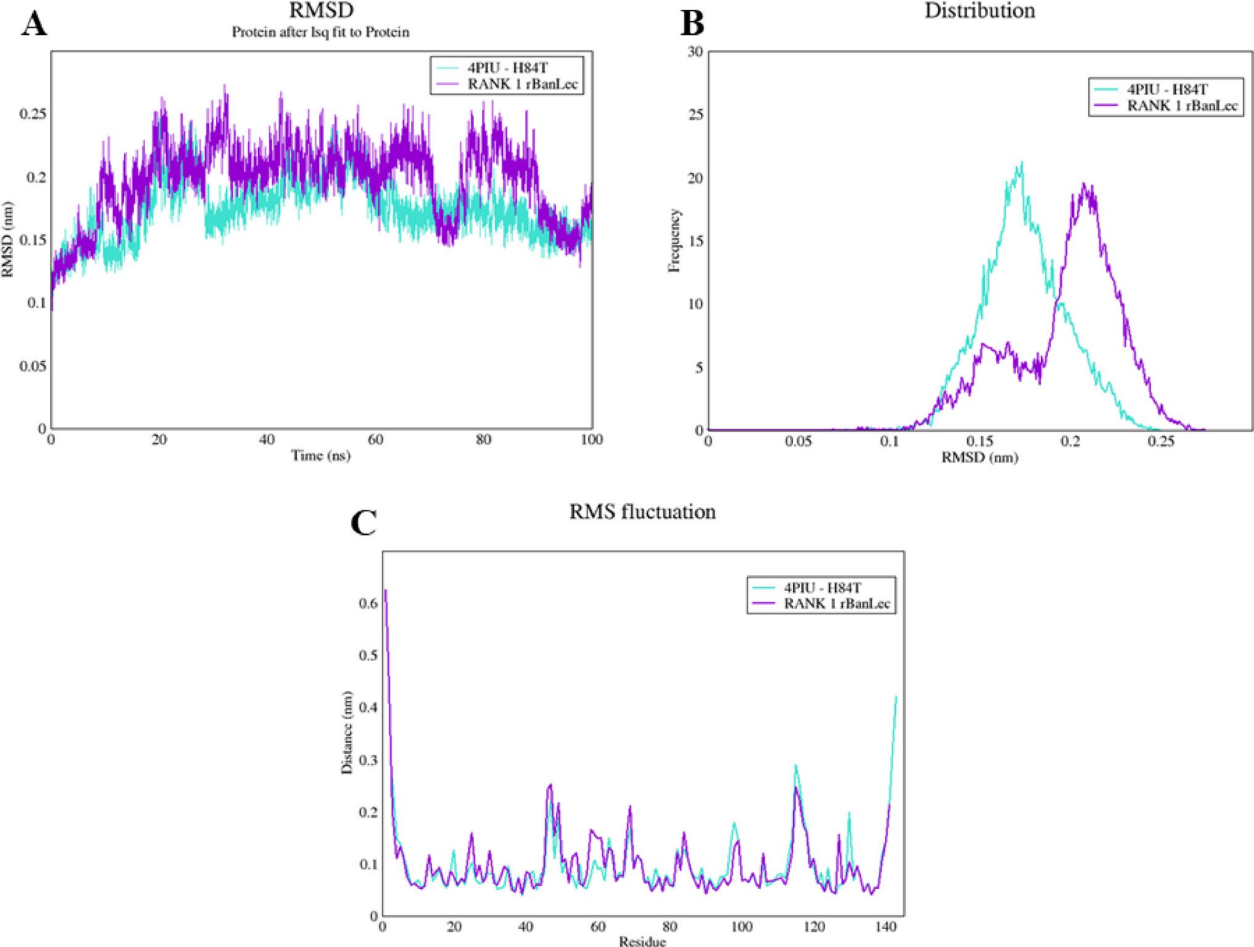
Molecular Dynamics Analysis Graphics. (**A**) RMSD analysis, indicating the structural stability of the proteins; (**B**) distribution analysis of the obtained values; and (**C**) RMSF analysis, showing the distance over which the amino acid residues fluctuated along the dynamic trajectory

The dynamics simulation of these proteins was performed using the GROMACS tool. For result analysis, this study selected RMSD (Root Mean Square Deviation), RMSF (Root Mean Square Fluctuation), and Distribution graphs (Fig. 8).

As observed in Fig. 8A, the lectin rBanLec-like (purple curve) shows a peak shifted to a higher RMSD (~ 0.20 nm), suggesting that this protein undergoes some structural variations at certain moments during the simulation, indicating greater flexibility. On the other hand, the lectin H84T-BanLec (light blue curve) displayed a lower RMSD (~ 0.15 nm), indicating that this protein remained more structurally stable. In Fig. 8B, the analysis of the conformational distribution of the proteins indicated that H84T-BanLec exhibited a uniform distribution pattern, while rBanLec-like displayed two distinct conformations in the molecular simulation environment. This behavior may explain the variation in the solubility of rBanLec-like observed in the results related to the expression of this protein, where it was expressed in both the soluble and insoluble fractions.

Finally, the RMSF was evaluated (Fig. 8C), which measures the fluctuations per residue over time, indicating which regions of the protein are more flexible or rigid. Both proteins show similar fluctuation patterns, with peaks in specific regions, suggesting that they share regions of higher flexibility. The small differences in the fluctuation peaks between the two proteins may indicate differences in the local stability of the structure. These differences also reveal variations in the movement of amino acids, especially in positions where there is a difference in the amino acid composition of the structures [28].

## Discussion

Despite their potential, the clinical use of lectins still faces barriers such as toxicity at high doses, mitogenicity, stability in biological environments, and specificity for target cells, for example. Another problem is that most of the known lectins with therapeutic potential are from natural sources and therefore their large-scale extraction is compromised. Therefore, much research is now focused on modifying lectins or using them as part of more complex systems (such as nanoparticles or conjugated antibodies).

To overcome some of these challenges, two lectins based on the BanLec lectin from *Musa acuminata* were designed and their synthetic genes cloned into expression vectors in *E. coli* [11]. The lectins rBanLec-like and H84T present differences in their primary sequences that could alter their activities. To understand how these mutations impact some important physicochemical parameters for the heterologous production of these proteins we produced these proteins in *E. coli* and further analyzed them.

This system was applied to the production of rBanLec-like and H84T-BanLec, allowing the comparison of the expression efficiency, yield, and biological activity of these proteins. However, the expression of lectins in bacteria often results in the formation of inclusion bodies, aggregates of denatured recombinant proteins resulting from failures in folding and formation of disulfide bridges [27]. A previous study by our research group demonstrated that rBanLec-like had a greater tendency to be expressed in the insoluble form [11]. However, in this study, it was possible to produce this lectin in *E. coli* in both soluble and insoluble forms (Fig. 1). It is important to highlight that in this previous study, the group evaluated the expression of rBanLec-like in the *E. coli* BL21 Star™ (DE3) strain. The replacement of the *E. coli* Star strain with the BL21(23)—Codon-Plus RIL® strain resulted in a significant increase in recombinant expression efficiency, exhibiting a higher protein production rate, as well as improved solubility and stability compared to the Star strain. This enhancement in solubility can be attributed to the presence of additional chaperone and disaggregation systems, such as DnaK, DnaJ, GrpE, and ClpB, which assist in the proper folding of recombinant proteins, preventing the formation of insoluble inclusion bodies and promoting protein stabilization in its soluble form (Nouri et al., 2016).

These in vitro data complement the in silico molecular dynamics simulation analysis, as rBanLec-like showed two distinct conformational distributions in the molecular simulation environment, indicating the folding could change (Fig. 8). Molecular modifications in a protein can cause disturbances that propagate to other regions, resulting in a redistribution of its dynamics. This phenomenon occurs as the protein responds to the entropy loss induced by the molecular changes [29]. This could explain the variation in the expression of rBanLec-like between the soluble and insoluble fractions. Otherwise, H84T-BanLec was fully expressed in the soluble form, a result that was also confirmed in silico. Computational analysis indicated a solid distribution point, demonstrating that the substitution of a single amino acid in the original BanLec sequence (histidine for threonine at position 84) maintained its stability and correct folding, in addition to improving its solubility (Fig. 1).These findings corroborate the data previously published by the research group at the University of Michigan [12, 15].

These observations are consistent with the strong influence of the three-dimensional structure and its modifications on protein solubility: well-organized conformations tend to expose hydrophilic residues to the aqueous medium, promoting dissolution, while poorly folded or denatured structures expose hydrophobic regions, favoring aggregation and precipitation. The studies carried out highlight the importance of investigating modified variants, since small changes in the sequence can significantly impact the properties of the protein, not only at the specific site of the substitution [30].

The ability of banana lectin to bind to mannose (especially high mannose) on tumor cells has been previously reported. Thus, the antitumor potential of rBanLec-like and H84T-BanLec lectins was evaluated in the HT-29 cell line. A study demonstrated that this strain exhibits a high content of high-mannose carbohydrates, making it a promising target for lectin-based therapeutic strategies using rBanLec-like and H84T-BanLec [31]. In addition to the two lectins, a low-activity banana lectin variant (D133G-BanLec) was used as a negative control. Our results demonstrated that both rBanLec-like and H84T-BanLec lectins had antiproliferative effects and efficiently induced cell death in the HT-29 cell line after 72 and 96 h of treatment (Fig. 2). It is important to highlight that, over 96 h, H84T-BanLec lectin exhibited a remarkable cell killing effect at all concentrations evaluated in this cell line (Supplementary material—Table S5). These results suggest that low concentrations can be tested initially. Morphologically, the cells responded to treatments with both lectins, changing their cell morphologies from flat to spherical, with a rounded shape and with the cell carpet destroyed, an effect accompanied by decreased cell viability.

Based on the results presented, the H84T-BanLec lectin showed a more pronounced cytotoxic effect on the HT-29 tumor cell line compared to rBanLec-like, especially at higher concentrations and longer incubation times. This difference can be mainly explained by the structural changes introduced in H84T-BanLec, which were originally designed to reduce the mitogenicity of wild-type BanLec, without compromising (or even enhancing) its affinity for specific carbohydrates [32]. Furthermore, the higher cytotoxic activity observed for H84T-BanLec may be related to its greater structural stability and solubility, characteristics that favor interaction with the cell surface and enable more efficient internalization or activation of pro-apoptotic pathways. On the other hand, although rBanLec-like also presents considerable cytotoxic activity, its interaction with glycans of the HT-29 cell line may be less efficient in the cellular context. Although molecular docking results suggested a higher affinity of rBanLec-like for mannose—reflected by a more favorable binding energy—, this in silico data does not directly translate into greater biological activity in vitro. This apparent discrepancy can be explained by several factors, such as differences in the accessibility of glycans on the cell surface, specificity for complex glycans, multivalence of the interaction, and other cellular mechanisms involved in cytotoxicity that are not directly related to mannose binding. Further, the affinity of H84T-BanLec is primarily for high mannose (5–9 mannoses on an asparagine) molecules, corroborating the molecular docking results. Therefore, it is understood that H84T-BanLec can trigger cell death signals more efficiently through mechanisms that rBanLec-like does not activate with the same intensity, considering that the binding affinity energy to the mannose containing complexes was lower than that to isolated D-mannose. Furthermore, it should be noted that the mutations promoted in BanLec-like were not directed to the amino acids that mediate the domains responsible for mitogenic activity, as was done in H84T-BanLec.

The fact that rBanLec-like was expressed in both the soluble and insoluble fractions suggests that part of the protein may have been produced in misfolded or aggregated forms, compromising its functionality. Consequently, only the soluble fraction would be fully active, reducing the effective amount of functional protein available to interact with cells. This factor may have negatively impacted its cytotoxic efficiency, since the dosage applied to cell cultures, even in the soluble fraction, disregards the possible structure change to inactive forms that may occur due to protein instability [33, 34].

## Conclusion

The advancement of molecular engineering has enabled the development of proteins with enhanced, attenuated, restricted, or even modified characteristics. In this study, we observed that, despite the modifications, both banana lectins, rBanLec-like and H84T- BanLec, maintained a structural pattern close to their native state, also preserving a high-affinity interaction with mannose, as well as structural stability in the molecular simulation environment. There is a high demand for the discovery of new biomolecules capable of performing strategic functions in various areas of biotechnology. A detailed understanding of the structure of proteins like lectins, and the interactions they establish with carbohydrates, is essential for a better understanding of biological products and the development of biotechnological processes and therapeutic applications. As technology continues to advance, bioinformatics tools are expected to continue to play a key role in protein characterization and the development of new therapies and treatments for a variety of diseases.

Finally, it is possible to state that H84T-BanLec continues to be one of the most promising lectins for therapeutic use in cancer and that complementary studies should be carried out to establish the potential of the new BanLec-like lectin.

## Supplementary Information

Below is the link to the electronic supplementary material.

Supplementary Material 1

## Data Availability

Research data are not shared.

## References

[1] Cagliari R, Kremer FS, Pinto LdaS (2018) *Bauhinia* lectins: biochemical properties and biotechnological applications. Int J Biol Macromol 119:811–820. 10.1016/j.ijbiomac.2018.07.156 30071232 10.1016/j.ijbiomac.2018.07.156

[2] Mishra A, Behura A, Mawatwal S et al (2019) Structure-function and application of plant lectins in disease biology and immunity. Food Chem Toxicol 134:110827. 10.1016/j.fct.2019.110827 31542433 10.1016/j.fct.2019.110827 PMC7115788

[3] Pinto S, Cardoso G, Schmitt F et al (2019) Heterologous expression and characterization of a new galactose- binding lectin from *Bauhinia* for *fi* cata with antiproliferative activity. Int J Biol Macromol 128:877–884. 10.1016/j.ijbiomac.2019.01.090 30721748 10.1016/j.ijbiomac.2019.01.090

[4] Van Damme EJM (2011) Lectins as tools to select for glycosylated proteins. Methods Mol Biol 753:289–297. 10.1007/978-1-61779-148-2_19 21604130 10.1007/978-1-61779-148-2_19

[5] Coelho LCBB, Silva PMDS, Lima VLDM et al (2017) Lectins, interconnecting proteins with biotechnological/pharmacological and therapeutic applications. Evid Based Complement Alternat Med. 10.1155/2017/1594074 28367220 10.1155/2017/1594074 PMC5359455

[6] Mckenna MK, Ozcan A, Brenner D et al (2023) Novel banana lectin CAR- T cells to target pancreatic tumors and tumor- associated stroma. J Immunother Cancer. 10.1136/jitc-2022-005891 36653070 10.1136/jitc-2022-005891 PMC9853244

[7] Pinho SS, Reis CA (2015) Glycosylation in cancer: mechanisms and clinical implications. Nat Rev Cancer 15:540–555. 10.1038/nrc3982 26289314 10.1038/nrc3982

[8] Mantuano NR, Natoli M, Zippelius A, Läubli H (2020) Tumor-associated carbohydrates and immunomodulatory lectins as targets for cancer immunotherapy. J Immunother Cancer 8:1–12. 10.1136/jitc-2020-001222 10.1136/jitc-2020-001222 PMC7537339 33020245

[9] Singh SS, Devi SK, Ng TB (2014) Banana lectin: a brief review. Molecules 19:18817–18827. 10.3390/molecules191118817 25407720 10.3390/molecules191118817 PMC6272006

[10] Hopper JTS, Ambrose S, Grant OC et al (2017) The tetrameric plant lectin BanLec neutralizes HIV through bidentate binding to specific viral glycans. Structure 25:773-782.e5. 10.1016/j.str.2017.03.015 28434916 10.1016/j.str.2017.03.015 PMC5556678

[11] de Camargo LJ, Maia MAC, Dos Santos Woloski R et al (2024) Characterization of a molecularly engineered Banlec-type lectin (rBTL). Mol Biotechnol 66:288–299. 10.1007/s12033-023-00752-9 37097521 10.1007/s12033-023-00752-9

[12] Swanson MD, Boudreaux DM, Salmon L et al (2015) Engineering a therapeutic lectin by uncoupling mitogenicity from antiviral activity. Cell 163:746–758. 10.1016/j.cell.2015.09.056 26496612 10.1016/j.cell.2015.09.056 PMC4641746

[13] Covés-Datson EM, King SR, Legendre M et al (2021) Targeted disruption of pi–pi stacking in Malaysian banana lectin reduces mitogenicity while preserving antiviral activity. Sci Rep 11:1–15. 10.1038/s41598-020-80577-7 33436903 10.1038/s41598-020-80577-7 PMC7804308

[14] Chan JFW, Oh YJ, Yuan S et al (2022) A molecularly engineered, broad-spectrum anti-coronavirus lectin inhibits SARS-CoV-2 and MERS-CoV infection in vivo. Cell Rep Med. 10.1016/j.xcrm.2022.100774 36195094 10.1016/j.xcrm.2022.100774 PMC9519379

[15] Covés-Datson EM, King SR, Legendre M et al (2020) A molecularly engineered antiviral banana lectin inhibits fusion and is efficacious against influenza virus infection in vivo. Proc Natl Acad Sci U S A 117:2122–2132. 10.1073/pnas.1915152117 31932446 10.1073/pnas.1915152117 PMC6995028

[16] Mosmann T (1983) Rapid colorimetric assay for cellular growth and survival: application to proliferation and cytotoxicity assays. J Immunol Methods 65:55–63. 10.1016/0022-1759(83)90303-4 6606682 10.1016/0022-1759(83)90303-4

[17] Mirdita M, Schütze K, Moriwaki Y et al (2022) ColabFold: making protein folding accessible to all. Nat Methods 19:679–682. 10.1038/s41592-022-01488-1 35637307 10.1038/s41592-022-01488-1 PMC9184281

[18] McGuffin LJ, Alharbi SMA (2024) ModFOLD9: a web server for independent estimates of 3D protein model quality. J Mol Biol 436:168531. 10.1016/j.jmb.2024.168531 39237204 10.1016/j.jmb.2024.168531

[19] Williams CJ, Headd JJ, Moriarty NW et al (2018) MolProbity: more and better reference data for improved all-atom structure validation. Protein Sci 27:293–315. 10.1002/pro.3330 29067766 10.1002/pro.3330 PMC5734394

[20] Abraham MJ, Murtola T, Schulz R et al (2015) Gromacs: high performance molecular simulations through multi-level parallelism from laptops to supercomputers. SoftwareX 1:19–25. 10.1016/j.softx.2015.06.001

[21] Jumper J, Evans R, Pritzel A et al (2021) Highly accurate protein structure prediction with AlphaFold. Nature 596:583–589. 10.1038/s41586-021-03819-2 34265844 10.1038/s41586-021-03819-2 PMC8371605

[22] Radjasandirane R, de Brevern AG (2024) AlphaFold2 for protein structure prediction: best practices and critical analyses. In: Lisacek F (ed) Protein Bioinformatics. Springer, New York, pp 235–252 10.1007/978-1-0716-4007-4_13 38995544

[23] Hollingsworth SA, Karplus PA (2010) A fresh look at the Ramachandran plot and the occurrence of standard structures in proteins. Biomol Concepts 1:271–283. 10.1515/BMC.2010.022 21436958 10.1515/BMC.2010.022 PMC3061398

[24] Park SW, Lee BH, Song SH, Kim MK (2023) Revisiting the Ramachandran plot based on statistical analysis of static and dynamic characteristics of protein structures. J Struct Biol 215:107939. 10.1016/j.jsb.2023.107939 36707040 10.1016/j.jsb.2023.107939

[25] Li J, Fu A, Zhang L (2019) An overview of scoring functions used for protein-ligand interactions in molecular docking. Interdiscip Sci 11:320–328. 10.1007/s12539-019-00327-w 30877639 10.1007/s12539-019-00327-w

[26] Paggi JM, Pandit A, Dror RO (2024) The art and science of molecular docking. Annu Rev Biochem 93:389–410. 10.1146/annurev-biochem-030222-120000 38594926 10.1146/annurev-biochem-030222-120000 PMC13198409

[27] Vohra RS, Murphy JE, Walker JH et al (2007) Functional refolding of a recombinant C-type lectin-like domain containing intramolecular disulfide bonds. Protein Expr Purif 52:415–421. 10.1016/j.pep.2006.11.012 17196395 10.1016/j.pep.2006.11.012

[28] Bibi S, Khan MS, El-Kafrawy SA et al (2022) Virtual screening and molecular dynamics simulation analysis of Forsythoside A as a plant-derived inhibitor of SARS-CoV-2 3CLpro. Saudi Pharm J 30:979–1002. 10.1016/j.jsps.2022.05.003 35637849 10.1016/j.jsps.2022.05.003 PMC9132386

[29] Gohlke H, Kuhn LA, Case DA (2004) Change in protein flexibility upon complex formation: analysis of Ras-Raf using molecular dynamics and a molecular framework approach. Proteins Struct Funct Bioinform 56:322–337. 10.1002/prot.20116 10.1002/prot.20116 15211515

[30] Ruiz FM, Scholz BA, Buzamet E et al (2014) Natural single amino acid polymorphism (F19Y) in human galectin-8: detection of structural alterations and increased growth-regulatory activity on tumor cells. FEBS J 281:1446–1464. 10.1111/febs.12716 24418318 10.1111/febs.12716

[31] Adlerberth I, Ahrné S, Johansson M-L et al (1996) A mannose-specific adherence mechanism in *Lactobacillus plantarum* conferring binding to the human colonic cell line HT-29. Appl Environ Microbiol. 10.1128/aem.62.7.2244-2251.1996 8779562 10.1128/aem.62.7.2244-2251.1996 PMC168005

[32] Covés-Datson EM, Dyall J, DeWald LE et al (2019) Inhibition of Ebola virus by a molecularly engineered banana lectin. PLoS Negl Trop Dis 13:1–20. 10.1371/journal.pntd.0007595 10.1371/journal.pntd.0007595 PMC6687191 31356611

[33] Singh A, Upadhyay V, Upadhyay AK et al (2015) Protein recovery from inclusion bodies of *Escherichia coli* using mild solubilization process. Microb Cell Fact. 10.1186/s12934-015-0222-8 25889252 10.1186/s12934-015-0222-8 PMC4379949

[34] Bhatwa A, Wang W, Hassan YI et al (2021) Challenges associated with the formation of recombinant protein inclusion bodies in *Escherichia coli* and strategies to address them for industrial applications. Front Bioeng Biotechnol. 10.3389/fbioe.2021.630551 33644021 10.3389/fbioe.2021.630551 PMC7902521

