# Supplementary material for: Comparative Analysis of Banana Lectins rBanLec-Like and H84T-BanLec: An In Silico and In Vitro Approach
Source: Protein J. 2026 Mar 19;45(2):318–31. doi: 10.1007/s10930-026-10326-8 (PMC13149608; doi:10.1007/s10930-026-10326-8)
Supplement: Supplementary file 1 — Supplementary Material 1 [file 10930_2026_10326_MOESM1_ESM.docx]

**Comparative Analysis of Banana Lectins rBanLec-like and H84T-BanLec: An *in Silico* and *in Vitro* Approach**

**^1^Guilherme Feijó de Sousa, ^1^Chrystian Nunes Gonçalves, ^1^Danillo de Oliveira Della Senta, ^1^Camila Garcia de Souza, ^1^Alice Calderipe de Lima, ^1^João Carlos Rodrigues, ^2^Maureen Legendre, ^2^David M. Markovitz, ^1^Luciano da Silva Pinto.**

*^1^Graduate Program in Biotechnology (PPGB), Bioinformatics and Proteomics Laboratory (BioPro Lab), Technological Development Center, Federal University of Pelotas;* *^2^Department of Internal Medicine, Division of Infectious Diseases, and Programs in Immunology, Cancer Biology, and Cellular and Molecular Biology University of Michigan, Ann Arbor, Michigan 48109, USA*

**Corresponding author:** Guilherme Feijó de Sousa (guilhermefeijodesousa@gmail.com)

**Supplementary Material**

**Tables:**

**Table S1.** Results of the stereochemical validation of the 3D model of the chimera. The ModFold9 software provides a quality score, while the Ramachandran plot shows the allowed regions for the positioning of the amino acids.

| Protein | Score  (ModFold9) | Confidence and *P-value* | Residues in more favored regions | Residues in favorable allowed regions | Residues in disallowed regions |  |
| --- | --- | --- | --- | --- | --- | --- |
| rBanLec-like | 0.9432 | 3.037E-5 | 134(96.40%) | 5(2.1%) | 2(1.5%) |  |
| H84T-BanLec | 0.9157 | 4.152E-5 | 135(97.12%) | 6(2.8%) | 0(0%) |  |

**Table S2.** Distribution of secondary structures for the rBanLec-like and H84T-BanLec proteins as determined by the SOPMA software.

| Secondary Structure | rBanLec (Residues) | rBanLec (%) | H84T (Residues) | H84T (%) |
| --- | --- | --- | --- | --- |
| Alpha helix (Hh) | 9 | 6.38% | 6 | 4.20% |
| 3_10_ helix (Gg) | 0 | 0.00% | 0 | 0.00% |
| Pi helix (Ii) | 0 | 0.00% | 0 | 0.00% |
| Beta bridge (Bb) | 0 | 0.00% | 0 | 0.00% |
| Extended strand (Ee) | 51 | 36.17% | 51 | 35.66% |
| Beta turn (Tt) | 0 | 0.00% | 0 | 0.00% |
| Bend region (Ss) | 0 | 0.00% | 0 | 0.00% |
| Random coil (Cc) | 81 | 57.45% | 86 | 60.14% |
| Ambiguous states (?) | 0 | 0.00% | 0 | 0.00% |
| Other states | 0 | 0.00% | 0 | 0.00% |

**Table S3.** Percentage of cell death of the HT-29 lineage in response to lectin treatments after 2 hours of incubation.

| **Concentration (µg/mL)** | **H84T-BanLec** | **Negative control** | **rBanLec-like** |
| --- | --- | --- | --- |
| 0 µg/mL | 0% | 0% | 0% |
| 6,25 µg/mL | 2.05% | 1.52% | 5.88% |
| 12,5 µg/mL | 7.44% | 4.1% | 7.66% |
| 25 µg/mL | 7.93% | 6.48% | 7.77% |
| 50 µg/mL | 14.95% | 8.15% | 7.93% |
| 100 µg/mL | 16.40% | 9.07% | 11.01% |
| 300 µg/mL | 18.83% | 12.52% | 11.93% |

**Table S4.** Percentage of cell death of the HT-29 lineage in response to lectin treatments after 72 hours of incubation.

| **Concentration (µg/mL)** | **H84T-BanLec** | **Negative control** | **rBanLec-like** |
| --- | --- | --- | --- |
| 0 µg/mL | 0% | 0% | 0% |
| 6,25 µg/mL | 41.32% | 3.30% | 36.5% |
| 12,5 µg/mL | 42.77% | 7.68% | 36.89% |
| 25 µg/mL | 47.76% | 11.75% | 43.31% |
| 50 µg/mL | 51.11% | 12.11% | 43.44% |
| 100 µg/mL | 51.50% | 12.63% | 45.26% |
| 300 µg/mL | 51.63% | 13.35% | 50.23% |

| **Concentration (µg/mL)** | **H84T-BanLec** | **Negative control** | **rBanLec-like** |
| --- | --- | --- | --- |
| 0 µg/mL | 0% | 0% | 0% |
| 6,25 µg/mL | 58.54% | 20.22% | 47.81% |
| 12,5 µg/mL | 61.07% | 27.10% | 49.13% |
| 25 µg/mL | 66.53% | 29.14% | 53.1% |
| 50 µg/mL | 69.83% | 29.57% | 53.1% |
| 100 µg/mL | 71.28% | 34.77% | 54.75% |
| 300 µg/mL | 76.00% | 35.59% | 62.6% |

**Table S5.** Percentage of cell death of the HT-29 lineage in response to lectin treatments after 96 hours of incubation.

**Figures**


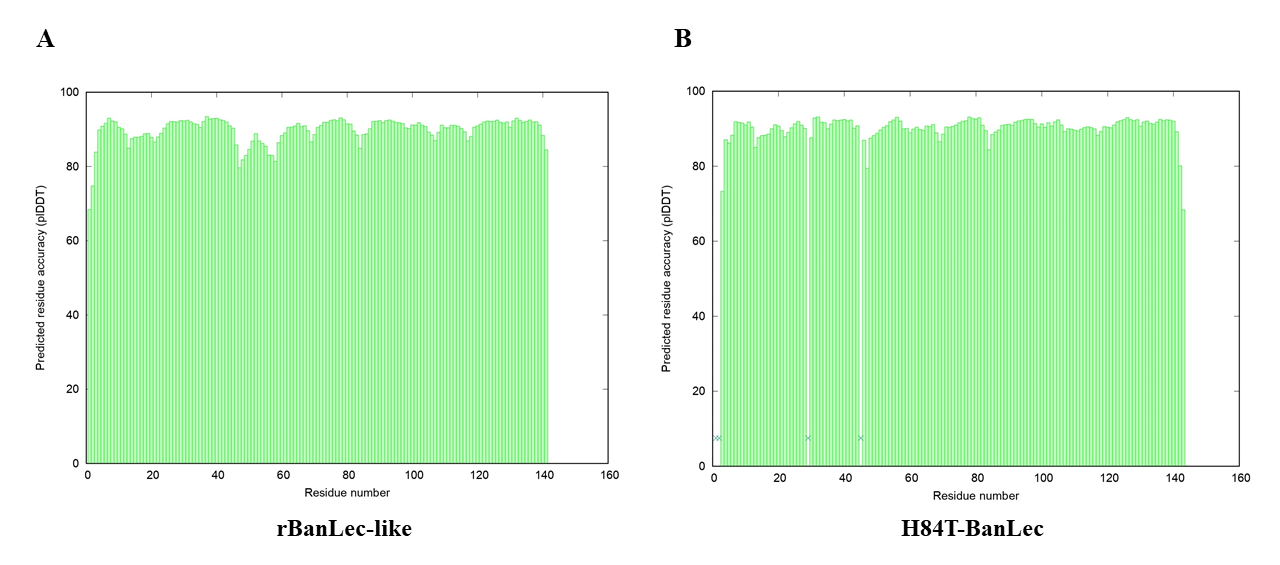


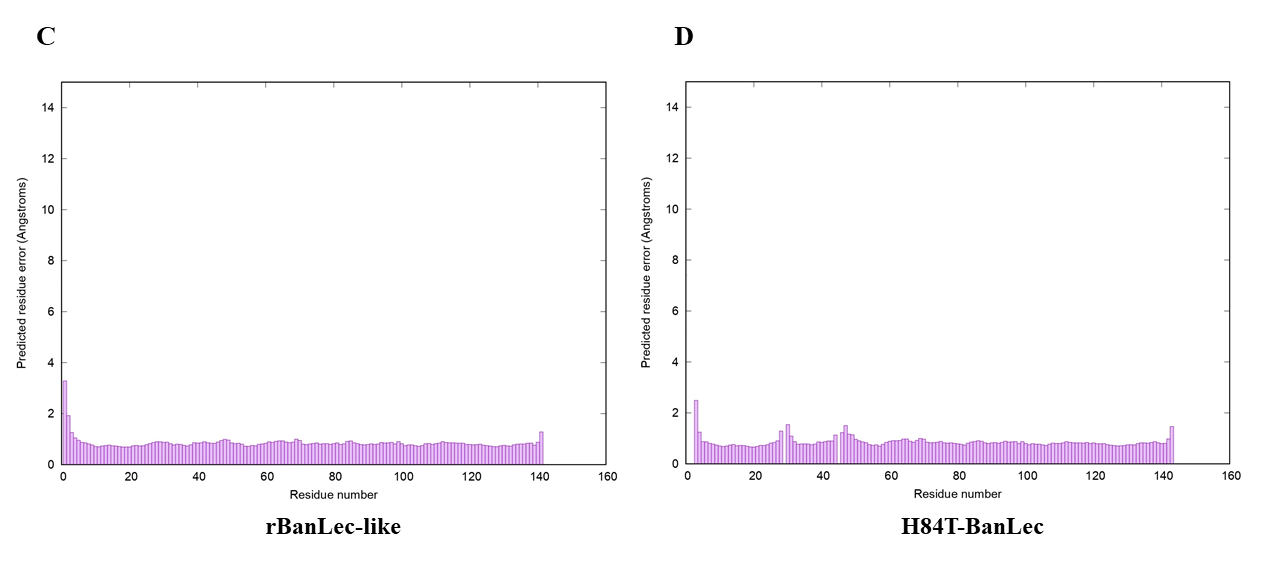


**Figure S1.** Quality analysis of the rBanLec-like and H84T-BanLec lectin models in the ModFold9 software. In (A) and (B), the predicted accuracy /per residue. In (C) and (D), the error per residue versus the number of residues.


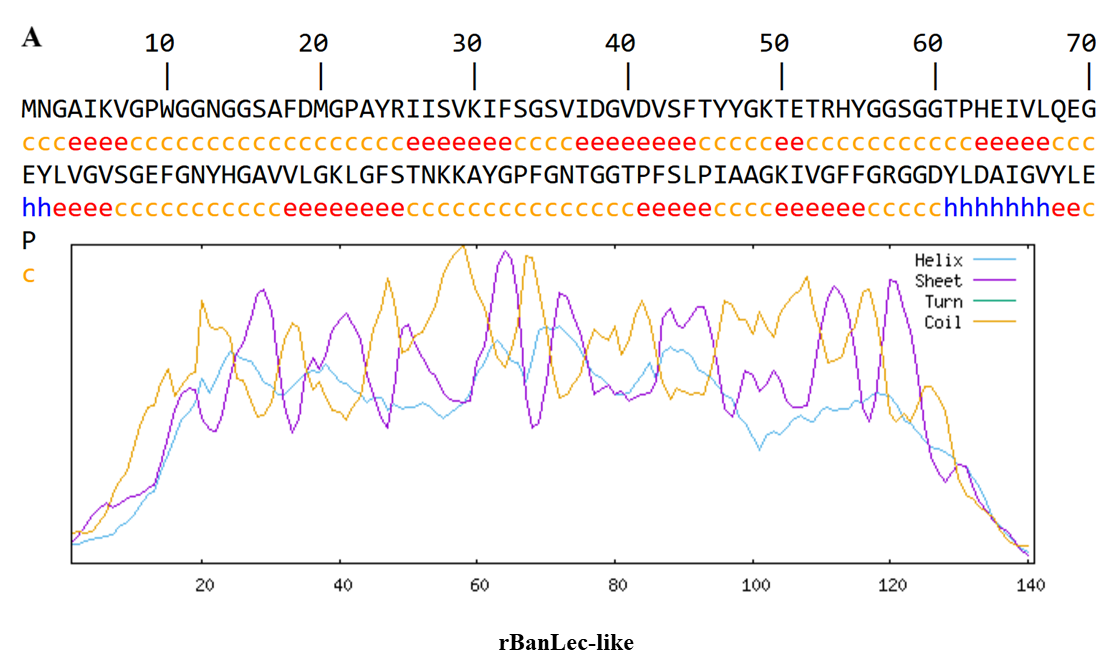


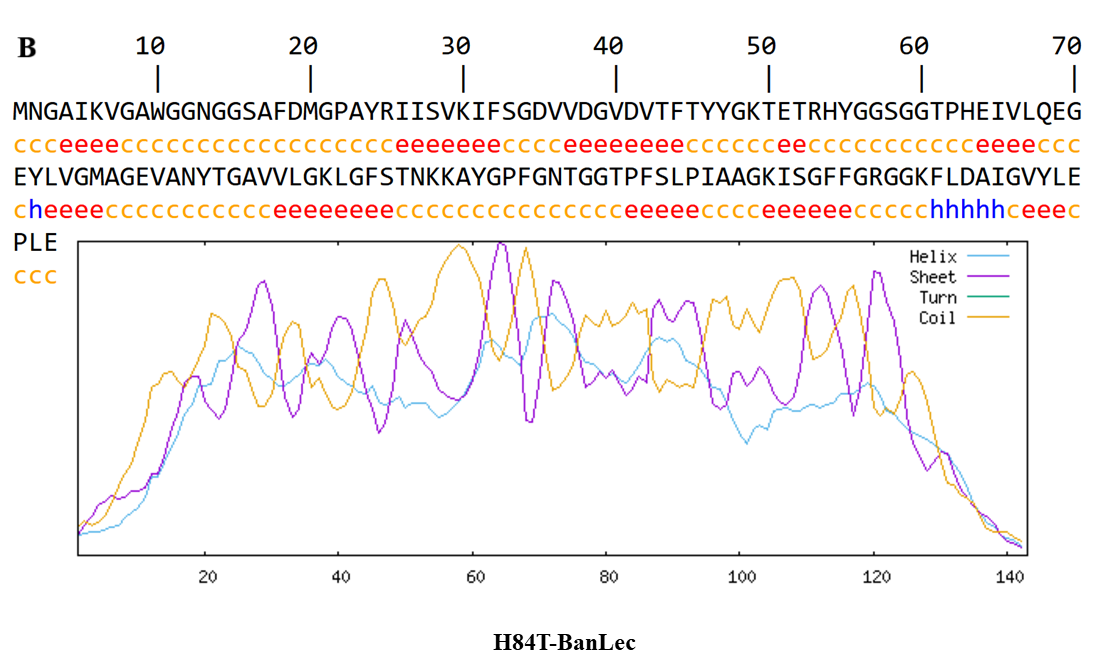


**Figure S2.** Prediction of the secondary structure of the rBanLec-like protein (A) and H84T-BanLec (B). The amino acid sequence is shown at the top, with the secondary structures indicated below: α-helices (blue), β-sheets (red), and coil regions (orange).

**Appendix:**

**Appendix 1.** Parameters used in grid box construction using AutoDock Tools.

| Protein | Center X | Center Y | Center Z | Box size |
| --- | --- | --- | --- | --- |
| rBanLec-like | -11.943 | 3.489 | -10.981 | 40×40×40 |
| H84T-BanLec | -10.755 | 1.968 | -9.983 | 40×40×40 |
